# Genetic Deletion of *Syndecan-4* Alters Body Composition, Metabolic Phenotypes, and the Function of Metabolic Tissues in Female Mice Fed A High-Fat Diet

**DOI:** 10.3390/nu11112810

**Published:** 2019-11-18

**Authors:** Maria De Luca, Denise Vecchie’, Baskaran Athmanathan, Sreejit Gopalkrishna, Jennifer A. Valcin, Telisha M. Swain, Rogerio Sertie, Kennedy Wekesa, Glenn C. Rowe, Shannon M. Bailey, Prabhakara R. Nagareddy

**Affiliations:** 1 Department of Nutrition Sciences, University of Alabama at Birmingham, Birmingham, AL 35294, USA; denisevecchie@gmail.com (D.V.); rogerio@uab.edu (R.S.); 2 Department of Biology, Ecology and Earth Sciences, University of Calabria, 87036 Rende, Italy; 3 Department of Surgery, Ohio State University, Columbus, OH 43209, USA; Baskaran.Athmanathan@osumc.edu (B.A.); gopalkrishna.sreejit@osumc.edu (S.G.); Prabhakara.Nagareddy@osumc.edu (P.R.N.); 4 Department of Pathology, University of Alabama at Birmingham, Birmingham, AL 35294, USA; javalcin@uab.edu (J.A.V.); telishaswain@uabmc.edu (T.M.S.); shannonbailey@uabmc.edu (S.M.B.); 5 Department of Biological Sciences, Alabama State University, Montgomery, AL 36104, USA; wekesa@alasu.edu; 6 Division of Cardiovascular Disease, University of Alabama at Birmingham, Birmingham, AL 35294, USA; glennrowe@uabmc.edu

**Keywords:** heparan sulfate proteoglycans, extracellular matrix, obesity, lipid profile, steatosis, insulin resistance

## Abstract

Syndecans are transmembrane proteoglycans that, like integrins, bind to components of the extracellular matrix. Previously, we showed significant associations of genetic variants in the *Syndecan-4* (*SDC4*) gene with intra-abdominal fat, fasting plasma glucose levels, and insulin sensitivity index in children, and with fasting serum triglyceride levels in healthy elderly subjects. An independent study also reported a correlation between *SDC4* and the risk of coronary artery disease in middle-aged patients. Here, we investigated whether deletion of *Sdc4* promotes metabolic derangements associated with diet-induced obesity by feeding homozygous male and female *Sdc4*-deficient (*Sdc4^-/-^*) mice and their age-matched wild-type (WT) mice a high-fat diet (HFD). We found that WT and *Sdc4^-/-^* mice gained similar weight. However, while no differences were observed in males, HFD-fed female *Sdc4^-/-^* mice exhibited a higher percentage of body fat mass than controls and displayed increased levels of plasma total cholesterol, triglyceride, and glucose, as well as reduced whole-body insulin sensitivity. Additionally, they had an increased adipocyte size and macrophage infiltration in the visceral adipose tissue, and higher triglyceride and fatty acid synthase levels in the liver. Together with our previous human genetic findings, these results provide evidence of an evolutionarily conserved role of *SDC4* in adiposity and its complications.

## 1. Introduction

The prevalence of obesity, characterized by an excess of white adipose tissue (WAT) mass, and the associated disease burden, are increasing worldwide [1]. Thus, the more we understand about the mechanisms underlying the link between excess body fat accumulation and adverse health effects, the more likely our chances of developing effective lifestyle and medical interventions to prevent and/or treat metabolic diseases. WAT is a complex multi-depot organ with the capability to expand or regress in response to excess or insufficient lipid storage caused by changes in energy balance [2]. One process that is required for WAT plasticity is the remodeling of the extracellular matrix (ECM) [3], which is an intricate network composed of two main classes of macromolecules, soluble proteoglycans and fibrous structural proteins (e.g., collagens, fibrillins, fibronectin, laminins) [4]. The ECM provides structural and anchoring support to the cells to stabilize cell morphology and tissue architecture, but it also regulates many aspects of the cell’s dynamic behavior by binding to cell-surface receptors, such as integrins and syndecans (SDCs) [4]. Because of its pivotal function, the ECM is constantly being modified by the resident cells during normal physiological processes, such as wound repair, angiogenesis, and adipose tissue remodeling [3,4]. However, excessive deposition of ECM components (e.g., fibrosis), as seen in obesity [2], can lead to organ dysfunction [5]. In this regard, the glycoproteins SDCs have been reported to play a key role in cardiac [6] and pulmonary [7] fibrosis.

SDCs belong to the family of heparan sulfate proteoglycans and are present on the cell surface of a wide range of invertebrate and vertebrate tissues [8,9]. Whereas invertebrates have only one *Sdc* gene and protein, that is expressed in most tissues, there are four genes (*SDC1, SDC2, SDC3*, and *SDC4*) and corresponding proteins in vertebrates [10]. Three of them (*SDC1, SDC2*, and *SDC3*) display a tissue-specific expression pattern, whereas the fourth, *SDC4,* is widely expressed in most adult tissues [11]. Across species, SDCs have a similar structural organization, consisting of an extracellular domain with attachment sites for glycosaminoglycans (GAGs) that is followed by a highly conserved transmembrane domain and a short cytoplasmic tail. The GAGs allow SDCs to directly interact with several ligands, including soluble growth factors, morphogens, cytokines, and ECM components [8]. However, it is through the binding sites of cytoskeleton proteins in the cytoplasmic tail that SDCs can control, independently and/or in synergy with the integrin-mediated signaling, fundamental cellular processes, including proliferation, adhesion, differentiation, fate determination, and migration [12,13,14,15].

Earlier work using *Sdc3* knockout mice first suggested important functions for SDCs in energy balance and obesity [16,17,18]. In later studies conducted in the fruit fly *Drosophila melanogaster*, we reported that young flies homozygous for a hypomorphic mutation of the *Sdc* gene displayed a lower whole-body metabolism than control flies [19]. Additionally, we demonstrated that knockdown of *Sdc* specifically in the fat body, the fly functional equivalent of both mammalian adipose tissue and liver [20], resulted in flies that had lower resting metabolic rates and ingested significantly less food than controls, but exhibited increased triglyceride (TG) levels [21]. In agreement with the findings in flies, we further identified significant associations of the single nucleotide polymorphism (SNP) rs1981429 mapping in the *SDC4* gene with increased intra-abdominal fat in healthy children [19] and higher levels of fasting plasma TG in healthy elderly individuals [22]. Remarkably, the *SDC4* rs1981429 https://www.sciencedirect.com/topics/neuroscience/polymorphism has also been found to increase the risk of coronary artery disease [23]. Taken together, these genetic observations suggest that *SDC4* might play a role in the relationship between excess abdominal adiposity and altered serum biochemical parameters, such as dyslipidemia, impaired fasting glucose, and liver dysfunction [24]. However, little is known about this issue. Here, we elicited obesity in homozygous male and female *Sdc4*-deficient (*Sdc4^-/-^*) mice and their age-matched WT mice by using a standard HFD treatment protocol [25,26]. The objective of the study was to evaluate the effects of *Sdc4* deficiency on body composition and energy balance components as well as cardiovascular disease-associated metabolic parameters in diet-induced obesity.

## 2. Materials and Methods

### 2.1. Animals and Husbandry

*Sdc4^-/-^* mice were previously generated on the C57BL/6 background and characterized by Echtermeyer et al. [27]. The mice were graciously provided by the Geir Christensen lab at the University of Oslo [7] after they had been repeatedly backcrossed to a C57BL/6J inbred background by Charles River (https://www.criver.com/microsites/jax-mice) in 2015. The average Percent Match of *Sdc4^-/-^* mice to the allelic profile of C57BL/6J was 99.6% and, therefore, the age-matched WT C57BL/6J mice were used as controls.

Beginning at seven weeks of age, female and male *Sdc4^-/-^* and WT control mice were randomly assigned to groups (*n* = 5–7/group) and maintained on an HFD (D12492, 60% kcal fat, 20% kcal protein, and 20% kcal carbohydrate, energy density: 5.21 kcal/g. Research Diets Inc., New Brunswick, NJ, USA) for 14 weeks. Mice were maintained in a temperature-controlled (22 °C) facility with a 12-h light/dark cycle and 50% humidity and given free access to food and water, except when the six-hour fasting blood specimens were obtained. Body weight and food intake were recorded at baseline and weekly for the first 8 weeks, and then at weeks 11, 12, and 14.

Following experimentation, all animals were euthanized with isoflurane using an adjusted flow rate, and gonadal WAT (gWAT) and liver tissues were harvested.

### 2.2. Body Composition, Activity, and Indirect Calorimetry

Body composition (total fat and lean mass), locomotor activity, and indirect calorimetry measurements were performed at the UAB Animal Physiology Core. Total fat and lean mass were assessed in unanesthetized mice using a noninvasive quantitative magnetic resonance imaging system (EchoMRI™ 3-in-1 v2.1; Echo Medical Systems, Houston, TX, USA), as previously reported [28]. Percent body fat and lean mass were calculated as [fat mass (or lean mass)/body weight] × 100.

Resting energy expenditure (REE), activity, and food intake were quantified using an eight-cage CaloSys indirect calorimetry system (TSE Systems, Inc., Chesterfield, MO, USA). To acclimate to the new environment, mice were individually kept in metabolic cages with ad libitum access to food and water for 48 h before measurements. Subsequently, O_2_ consumption, CO_2_ production, food intake, and spontaneous locomotor activity were continuously measured for 24 h. REE was determined as the average of the three lowest 18 min-intervals, with at least 1 h in between intervals, as previously reported [29]. Locomotor activity was monitored by a multidimensional infrared light beam system surrounding each cage.

### 2.3. Plasma Analyses and Glucose Tolerance Test

Blood samples were collected from the central tail artery after six hours fasting that started at 7:00 a.m. (Zeitgeber Time, ZT 1; ZT 0 = lights on). Total cholesterol and TG levels in plasma were measured using colorimetric assays (FUJIFILM Wako Diagnostics USA Corporation, Richmond, VA, USA). Plasma insulin was measured using an ELISA kit (cat. No 80-INSMS-E01, ALPCO Immunoassays, Salem, NH, USA) per manufacturer’s instructions. Blood glucose was measured directly from the tail tip using a One Touch Ultra 2 glucose monitoring system (Lifescan, Johnson & Johnson, New Brunswick, NJ, USA).

For the oral glucose tolerance test (OGTT), mice were gavaged with 25% D- (+) -glucose after six hours of fasting at a dose of 2 g/kg. Blood samples were taken at 0, 15, 30, 60, and 120 min for the measurement of plasma glucose and insulin concentrations. The whole-body insulin sensitivity index (ISI) was derived from the OGTT using Matsuda and DeFronzo’s equation: 10,000/square root of [fasting glucose × fasting insulin] × [mean glucose × mean insulin during OGTT] [30].

### 2.4. Histological Analyses

gWAT pads were excised and fixed in 10% neutral buffered formalin. Tissues were embedded into paraffin blocks and 5 µm thick sections were prepared for each sample at the UAB Comparative Pathology Laboratory, where the hematoxylin and eosin (H&E) staining was also performed. Staining of Type I and III collagen fibers was performed with a Picro-Sirius red staining kit (cat. No VB-3017, VitroVivo Biotech, Rockville, MD, USA) according to the manufacture’s protocol. Immunohistochemistry for macrophage marker F4/80 and Type VI collagen (COL6) alpha 1 fibers was performed using the 3,3’-Diaminobenzidine (DAB) horseradish peroxidase (HRP) substrate (cat. No 8059, Cell Signaling Technology, Danvers, MA, USA) and the protocol reported in [31]. Sections were incubated overnight at 4 °C with rabbit anti-F4/80 monoclonal antibody (cat. No 70076, Cell Signaling Technology, Danvers, MA, USA) or rabbit anti-COL6 alpha 1 polyclonal antibody (cat. No NB120-6588, Novus Biologicals, Centennial, CO, USA) and incubated at room temperature for 30 min with the SignalStain^®^ Boost Detection Reagent (cat. No 8114, Cell Signaling Technology, Danvers, MA, USA).

Digital images were acquired with a microscope Leica DMRB using a color camera and XnView software. Five medium-power field (10 ×) images were acquired at regular spatial intervals from three to four animals in each group. Adipocyte area was determined by measuring approximately 100 cells per animal using software Motic-Images Plus 2.0 [32]. Percent area of staining for picro-Sirius red, F4/80, and COL6 was quantified using ImageJ software.

### 2.5. Hepatic TG Levels

Total lipids were extracted from livers using a modified Folch extraction procedure [33]. Frozen liver (20–25 mg) was homogenized in chloroform–methanol (2:1), incubated at room temperature, and phases were separated by the addition of sulfuric acid (0.05%). The lower organic phase was collected and TG content was measured using the L-Type Triglyceride M colorimetric assay (FUJIFILM Wako Diagnostics USA Corporation, Richmond, VA, USA) as described in [34].

### 2.6. RNA Isolation and Gene Expression

Total RNA was extracted from frozen tissues (liver and gWAT) using TRI-Reagent (Sigma-Aldrich). Isolated RNA was DNase treated using a DNase Treatment Kit (Life Technologies) and DNase-treated RNA (1 µg) was converted to cDNA using the High-Capacity cDNA Reverse Transcription kit (Thermo Fisher Scientific, Waltham, MA, USA). Quantitative PCR was performed in triplicate using SYBR Green Master Mix (Bio-Rad). Relative expression levels were determined using the 2^−ΔΔCt^ formula [35] by normalizing the gene of interest to the following housekeeping genes: *Hypoxanthine-guanine phosphoribosyl transferase* (*Hprt*), *TATA-box binding protein* (*Tbp*), and *Actin, beta* (*Actb*). Primers used in the study are reported in Supplementary Table S1.

### 2.7. Western Blotting Analysis

Liver homogenates were prepared in 0.25 M sucrose buffer, pH 7.4, supplemented with protease and phosphatase inhibitor cocktails (Millipore Sigma, St. Louis, MO, USA). Equal amounts of protein were separated by sodium dodecyl sulfate–polyacrylamide gel electrophoresis and transferred onto PVDF membrane (Thermo Fisher Scientific, Rockford, IL, USA), blocked in 5% BSA, and incubated with 1:5000 rabbit β-Actin (cat No 4967, Cell Signaling Technology, Danvers, MA, USA) and 1:1000 rabbit FASN (cat No 3180, Cell Signaling Technology, Danvers, MA, USA).

### 2.8. Statistical Analysis

Two-way analysis of variance (ANOVA) with repeated measures was performed to compare percent changes in body weight over the intervention period (variable time), with sex and genotype and their interactions, with time included in the model. Mauchly’s test was used to assess the assumption of sphericity, and Greenhouse–Geisser correction was used for violations of this assumption. Two-way ANOVA models were run for energy balance phenotypes and metabolic parameters, with genotype, sex, and genotype-by-sex interaction terms included in the model. A log^10^ transformation was applied to the data that did not meet the assumption of normality. The Tukey test for post-hoc pairwise comparisons was implemented to assess significant differences between groups. The non-parametric Kolmogorov–Smirnov test was used to test the differences between cumulative distributions of adipocyte size. The non-parametric Wilcoxon signed-rank test was used to compare differences between female *Sdc4^-/-^* and WT mice for the other experimental assays. Statistical analyses were performed with SAS version 9.4 (SAS Institute, Cary, NC, USA). A significant level of 0.05 was used throughout the study.

### 2.9. Ethics Approval 

All protocols were approved by the Institutional Animal Care and Use Committee (IACUC-20126) at the University of Alabama at Birmingham and followed the Guide for the Care and Use of Laboratory Animals.

## 3. Results

### 3.1. Sdc4 Deficiency Increases Percent Body Fat Mass and Exacerbates Metabolic Complications in Obese Female Mice but Not in Males 

**Figure 1:**
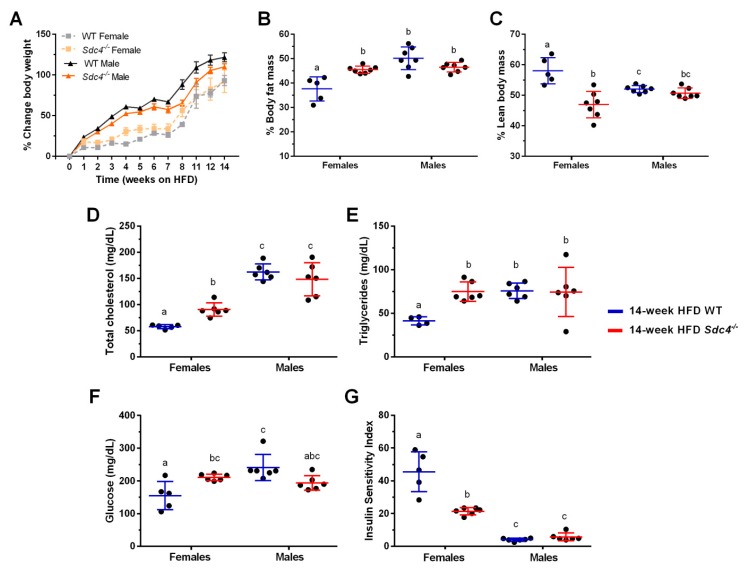
Female-specific effects of *Sdc4* deficiency on body composition and metabolic phenotypes in high-fat diet (HFD)-induced obesity. (**A**) Both female and male mice gained weight when fed an HFD for 14 weeks, independent of genotype. (**B–G**) Compared to WT mice, only female *Sdc4^-/-^* mice had more % body fat mass (Panel **B**), less % lean body mass (Panel **C**), higher levels of fasting plasma total cholesterol (Panel **D**), triglycerides (Panel **E**) and glucose (Panel **F**), and lower whole body insulin sensitivity (Panel **G**) following the 14-week diet intervention. Data represent means for *n* = 5–7 animals of raw data. Error bars represent standard errors. Significant comparisons were determined by post hoc Tukey’s tests at *p* < 0.05 and are indicated by different letters.

Consumption of HFD leads to obesity and its metabolic sequelae in laboratory mice, such as those from the C57BL/6J inbred strain [25,26]. Further, male obese have been reported to be more susceptible than females to the effects of HFD on weight gain and metabolic alterations [36]. Consistent with these observations, we observed a significant increase in weight gain over the 14-week treatment, with males on average gaining more weight than females (Figure 1A, Table 1). However, no effect of the genotype was detected in our analysis (Figure 1A, Table 1), indicating that mice with a deficiency of *Sdc4* gained the same amount of weight as the control mice when fed a diet containing high-fat content.

We previously reported significant associations between *SDC4* rs1981429 and variation in intra-abdominal adipose tissue and lean tissue mass in a cohort of American children [19]. Specifically, we found that children homozygous for the less common allele had more visceral fat and less lean tissue mass than those carrying at least one copy of the other allele [19]. Additionally, growing evidence from many species suggests the involvement of SDCs in skeletal muscle development [37]. Based on these observations, we decided to assess body composition after the 14-week dietary treatment period.

Notably, two-way ANOVA revealed significant effects of sex and genotype on both percent body fat mass (%BFM) and percent lean body mass (%LBM) (Table 2) but also a significant interaction between genotype and sex, indicating that the genotype significance was driven by the sex of the animal (Table 2). As shown in Figure 1, while no differences were observed in males, HFD-fed females *Sdc4^-/-^* mice displayed on average higher (25%) %BFM (Figure 1B) and less (19%) %LBM (Figure 1C) than WT.

We then investigated whether the increase in %BFM observed in female *Sdc4^-/-^* mice was accompanied by alterations in metabolic parameters that are associated with increased T2D and cardiovascular risk. There was no effect of genotype on fasting plasma insulin levels (Table 2). However, significant interactions between genotype and sex were observed for total cholesterol, TG, and glucose levels, and for whole-body ISI (Figure 1, Table 2). HFD-fed female *Sdc4^-/-^* mice exhibited higher levels of fasting plasma total cholesterol (36%) (Figure 1D) and TG (46%) (Figure 1E), as well as a significant increase (27%) in fasting blood glucose levels (Figure 1F). This change in glucose levels mirrored a significant decrease (53%) in the whole-body ISI (Figure 1G). Together, these data demonstrate that the effects of *Sdc4* deficiency on body composition and cardiometabolic phenotypes are sexually dimorphic in mice challenged with an HFD.

### 3.2. Sdc4 Deficiency Reduces Food Intake in Obese Mice, Independent of Sex

Next, we sought to examine whether changes in energy balance might explain the increased %BFM in HFD-fed female *Sdc4^-/-^* mice. A two-way ANOVA analysis detected significant effects of sex and genotype on food intake (Table 3). Notably, contrary to our assumption, we found that *Sdc4^-/-^* mice ate on average statistically significant less (18%) food than WT, independent of sex (Table 3).

On the other hand, there were no significant differences in spontaneous locomotor activity or REE between *Sdc4^-/-^* and WT mice (Table 3). Thus, our results suggest that *Sdc4* deficiency decreases food intake in HFD-induced obesity.

### 3.3. Sdc4 Deficiency Induces Adipocyte Hypertrophy and Macrophage Infiltration into Visceral Adipose Tissue of Obese Female Mice, but Not Fibrosis

**Figure 2:**
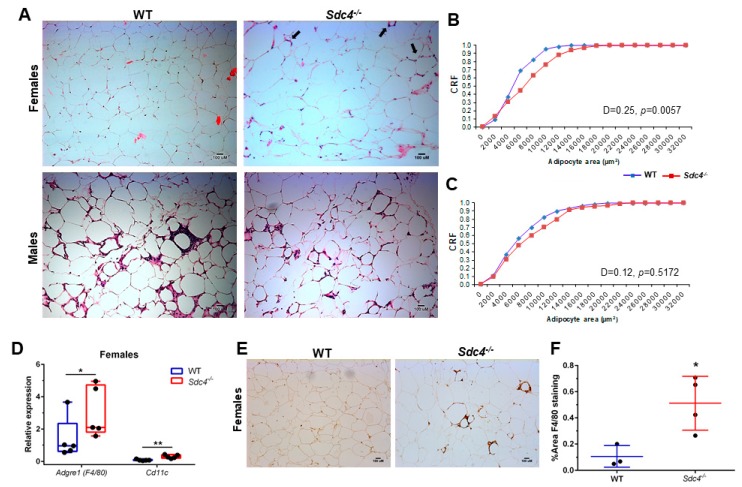
*Sdc4* deficiency leads to an increase in visceral adipocyte size and macrophage infiltration in female mice fed an HDF for 14 weeks. (**A**) Representative haematoxylin and eosin of gonadal WAT (gWAT) sections from mice fed an HFD for 14 weeks. Black arrows in top right panel depict cell infiltration. (**B**,**C**) Cumulative relative frequencies (CRF) distribution of adipocyte size from female (Panel **B**) and male (Panel **C**) mice (100 cells per animal; *n* = 3–4). (**D**) Gene expression levels were measured by qPCR using mRNA isolated from gWAT. Box and whiskers plots denote individual data points, separated by a line representing the group median. Each individual value is plotted as a dot superimposed on the boxplots (*n* = 5). Transcript levels of each target gene were normalized to *Hprt*, *Actb*, and *Tbp*. (**E**) Representative images of immunohistochemical staining for F4/80 protein. (**F**) Data represent means for *n* = 3–4 animals, with five not overlapping images taken per animal. Error bars represent standard errors. In panels **D** and **F**, * *p* < 0.05 and ** *p* < 0.01, compared to WT.

To assess whether the higher levels of total body fat mass in female *Sdc4^-/-^*mice may reflect increased visceral adiposity, as seen in humans [19], we quantified adipocyte size from H&E-stained gWAT sections of both female and male WT and *Sdc4^-/-^*mice. We found that female *Sdc4^-/-^*mice had significantly greater adipocyte size than female WT animals (Figure 2A,B). No significant differences were observed in males (Figure 2A,C).

**Figure 3:**
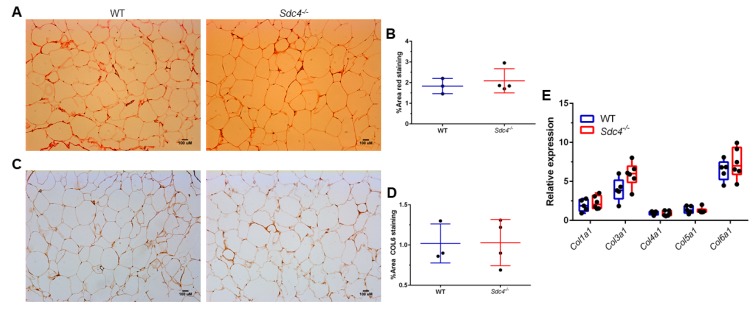
*Sdc4* deficiency does not affect collagen levels in gonadal WATs isolated from female mice fed an HDF for 14 weeks. (**A**,**B**) Representative images of picrosirius red staining (Panel **A**) with quantification (Panel **B**). (**C**,**D**) Representative images of immunohistochemical staining for Collagen VI, alpha 1 (COL6) (Panel **C**) with quantification (Panel **D**). In panels **B** and **D**, data represent means for *n* = 3–4 animals, with five not overlapping images taken per animal. Error bars represent standard errors. (**E**) Gene expression levels were measured by qPCR using mRNA isolated from gWAT. Box and whiskers plots denote individual data points separated by a line representing the group median. Each individual value is plotted as a dot superimposed on the boxplots. Transcript levels of each target gene were normalized to *Hprt*, *Actb*, and *Tbp*.

Cell infiltration was also seen to a greater degree in H&E gWAT sections from HFD-fed female *Sdc4^-/-^*mice than from female WT mice (Figure 2A, top right panel), suggesting a higher infiltration of leukocytes. To assess this idea, we measured the transcript levels of *Adgre* (encoding the macrophage population marker F4/80) and *Cd11c* (encoding the dendritic cell marker CD11c) in gWAT from female WT and *Sdc4^-/-^*mice. As predicted, there was a statistically significant higher expression level of both *Adgre* and *Cd11c* in female *Sdc4^-/-^*mice compared to female controls (Figure 2D). This finding was further corroborated by IHC staining of an F4/80 protein amount, which was found to be significantly higher in female *Sdc4^-/-^*mice (Figure 2E,F). To determine whether the increased adiposity was accompanied by fibrosis, we performed picrosirius red staining for Type I and III collagen identification, and IHC staining for COL6 in gWAT tissues. There was no difference in red staining (Figure 3A,B) or IHC staining of COL6 (Figure 3C,D) between female WT and *Sdc4^-/-^*mice. qPCR analysis of collagen gene transcript levels confirmed the staining results (Figure 3E). Together, these findings suggest that the disruption of *SDC4* in female mice promotes visceral hypertrophy and macrophage infiltration with caloric excess.

### 3.4. Sdc4 Deficiency Leads to Increased TG Content and FASN Levels in the Liver of Obese Female Mice

**Figure 4:**
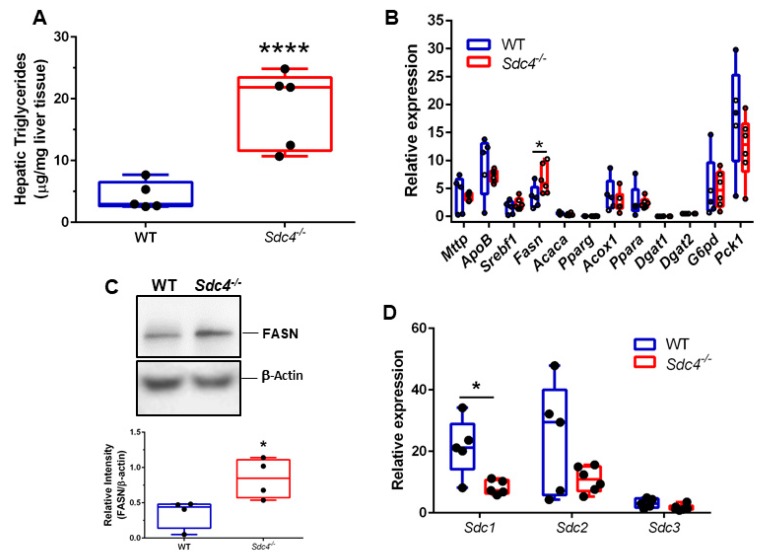
*Sdc4* deficiency leads to higher levels of hepatic triglycerides and FASN and lower *Sdc1* transcript levels in female mice fed an HFD for 14 weeks. (**A**–**D**) Box and whiskers plots denote individual data points for hepatic triglycerides (Panel **A**), transcript levels of lipid and glucose metabolism genes (Panel **B**), FASN protein levels (Panel **C**), and transcript levels of *Sdc* genes (Panel **D**), separated by a line representing the group median. Each individual value is plotted as a dot superimposed on the boxplots. In panel **C**, representative Western blotting for FASN, with β-actin used as loading control. In panels **B** and **D**, transcript levels of each target gene were normalized to *Hprt*, *Actb*, and *Tbp*. In all panels, * indicates. *P* < 0.05 and **** *p* < 0.0001 compared to WT mice.

Obesity predisposes to hepatic steatosis, a condition characterized by the deposition of lipid droplets in the liver [38]; therefore, we next measured hepatic TG in HFD-fed female *Sdc4^-/-^* and WT mice. We found that female *Sdc4^-/-^* mice displayed markedly increased (77%) levels of hepatic TG compared to WT (Figure 4A). To explore whether *Sdc4* deficiency elicits changes in hepatic metabolism that might explain the increased hepatic and plasma TG levels, as well as the increased plasma glucose levels, in female *Sdc4^-/-^* mice, we next quantified transcript levels of key genes involved in hepatic lipid and glucose metabolism. Only *Fasn* transcript levels were significantly higher in female *Sdc4^-/-^* mice than in control mice (Figure 4B). The increase in *Fasn* transcriptional levels was reflected in a significant increase in FASN protein levels (Figure 4C). These data suggest a role for *SDC4* in hepatic FASN regulation and, therefore, liver fat content under HFD conditions.

### 3.5. Sdc4 Deficiency Leads to Decreased Sdc1 Transcript Levels in the Liver of Obese Female Mice

It has been reported that the deletion of one SDC can induce a compensatory upregulation of other SDC paralogs [39]. As such, we further investigated whether the expression levels of *Sdc1*, *Sdc2*, and *Sdc3* were changed in the liver of obese female *Sdc4^-/-^*mice compared to controls. While no differences were observed for *Sdc2* and *Sdc3* transcript levels between female *Sdc4^-/-^*mice and WT mice, we found that the expression of *Sdc1* levels was approximately 60% lower in obese female *Sdc4^-/-^*mice (Figure 4D). Notably, no differences between female *Sdc4^-/-^* mice and WT mice were found in the expression levels of *Sdc1*, *Sdc2*, *Sdc3,* or *Fans* in gWAT (Figure S1). SDC1 is the most abundant SDC in the liver, where it plays a critical role in the clearance of triglyceride-rich lipoproteins [40]. Specifically, loss of hepatic *SDC1* results in mice with impaired very-low-density lipoprotein metabolism and hypertriglyceridemia [40]. Thus, this observation and our finding suggest that reduced levels of *Sdc1* expression might mediate the effects of *Sdc4* deficiency on hypertriglyceridemia.

## 4. Discussion

We previously reported pleiotropic associations of human *SDC4* variants with inter-individual variability in body composition (rs1981429), fasting plasma glucose levels (rs4599), and insulin sensitivity (rs2267871) in children [19]. In the present study, we confirmed these findings in obese mice lacking *Sdc4*. In addition, we revealed that the effects of the *Sdc4* null mutation on these phenotypes are specific to female mice. Due to the small sample size, *SDC4* SNP-by-sex interaction terms were not included in the association analyses performed in our earlier human studies [19,22]. Furthermore, those studies did not explore the effect of potential interactions between dietary pattern and *SDC4* SNPs on the above phenotypes. Our finding in mice motivates additional work in larger human populations to investigate whether *SDC4* can be used as a biomarker for obesity risk in women.

Previous work by Stubbins et al. [26] has provided evidence that C57BL/6J male mice gain more weight and have higher adiposity than females after 10 weeks of consuming an HFD. The authors also showed that estrogen protects females from developing insulin resistance when exposed to the obesogenic effect of an HFD, most likely because of the estrogen-mediated protective effect against liver steatosis and adipocyte inflammation [26]. We replicated the sex differences in adiposity and metabolic phenotypes in WT C57BL/6J mice but we also found that, compared to female WT, HFD-fed female *Sdc4^-/-^* mice exhibited a higher %BFM and developed a worse metabolic asset, such as increased levels of plasma TG and glucose, and reduced insulin sensitivity. In addition, they displayed increased adipocyte hypertrophy and macrophage infiltration into visceral WAT and hepatic TG levels. No differences were observed in collagen deposition between female *Sdc4* mutant mice and WT mice. This finding is consistent with the most recent report that the appearance of collagen deposition follows WAT leukocyte infiltration during diet-induced obesity progression in C57BL/6J mice [41]. Based on this recent study, fibrosis is widespread at 16 weeks of an HFD [41]; therefore, it is likely that differences were not detected because our dietary intervention was ended at 14 weeks. Considering the multicellular composition of WAT and the involvement of several cells, such as macrophages, adipocyte progenitors, and mature adipocytes, in obesity-induced WAT fibrosis [42], as well as the ubiquitous expression pattern of *SDC4* [11], we cannot, however, rule out the idea that compensatory mechanisms prevented the development of gWAT fibrosis in the obese female *Sdc4^-/-^* mice.

A limitation of our study is that accurate measurements of body composition and components of energy balance were not performed at baseline and throughout the intervention. However, analyses of the data collected after the 14-week intervention period showed that HFD-fed *Sdc4^-/-^* mice ingested less food than WT mice and that the effect of the mutation on food intake was independent of sex. Growing evidence indicates that SDCs promote food intake by binding, through their GAGs, to the agouti-related peptide (AgRP), thereby improving its efficacy in the hypothalamic feeding circuit [17,18,43,44,45]. Earlier studies argued for the SDC3 protein as the *sole* mediator of the process, since it is highly expressed in the hypothalamus [43]. More recently, elegant work performed by Palomino and colleagues [45] reported the presence of sites in the AgRP protein structure that are critical for its orexigenic effects and bind with high affinity to GAGs without specificity. Our finding aligns with this observation and suggests that SDC4 might be involved in the Agouti/Agrp-melanocortin system in response to an HFD challenge.

The observation that female *Sdc4^-/-^* mice displayed increased visceral adiposity while eating less food without showing changes in REE and locomotor activity suggests a potential cell-autonomous role for SDC4 in adiposity. Cell culture studies have demonstrated that the SDC4 protein is expressed in mature white adipocytes [31], and a possible hypothesis is that genetic deletion of *Sdc4* promotes hypertrophy through activation of an adipocyte-mediated molecular mechanism. WAT displays a large number of significant differences in gene expression between the sexes in both mice [46] and humans [47]. Among the sexually dimorphic genes identified in the mouse WAT, there are those encoding actinins, cadherins, and calcium channel subunits [46], which are molecules involved in cell–matrix and cell–cell adhesions and therefore likely to functionally interact with SDC4 [48]. The main limitation of this study is that mature adipocytes were not separated from the stromal vascular fraction. Therefore, we could not perform experiments for the molecular characterization of *Sdc4* deficiency in adipocytes that may pinpoint to potential sex-specific mechanism/s through which SDC4 regulates adiposity. Further investigations using an adipose tissue-specific *Sdc4* knockout mouse model are currently underway in our laboratory to define the sexually-dimorphic role of SDC4 in adipocyte function. Recently, Scherer and colleagues reported that an endothelial-to-adipocyte extracellular vesicle axis exists in WAT [49]. Given that SDC4 regulates exosome biogenesis [50] and is expressed in endothelial cells [51], an alternative hypothesis is that its deficiency might impair the cross-talk of extracellular vesicles between endothelial cells and adipocytes, and consequently adipocyte lipid metabolism. Moreover, estrogen has been shown to regulate the profile of microRNAs transported within exosomes in humans [52] and this might explain the sex-specific differences seen in our study. Yet, it is well-recognized that circulating TG are the major source of fatty acids entering the adipose tissue in both humans and mice [53], hence, we cannot exclude the possibility that the increased visceral adiposity in female *Sdc4^-/-^* mice may be due to their higher levels of circulating TG [24].

HFD-induced obesity promotes steatosis in mice, and increased expression levels of hepatic *Fasn* have been previously reported in murine models of hepatic steatosis [54]. Consistent with this, we further demonstrated that obese female *Sdc4^-/-^* mice had increased transcript levels of hepatic *Fasn,* a downstream target of sterol-regulatory element-binding factor-1 (SREBF-1), which, in turn, is a transcription factor that controls lipid homeostasis [55]. Notably, the increase in *Fasn* was not accompanied by an increase in the expression levels of *Srebf1* or *Acaca*, an additional SREBF-1 target-gene, suggesting that the expression of *Fasn* is induced via an alternative pathway. The liver X receptors (LXRs) are nuclear factors activated by oxysterols, which are intermediates or end-products of cholesterol metabolism and regulate cholesterol, bile acid, and lipoprotein metabolism [56]. The LXR signaling pathway controls *Fasn* expression through distinct but complementary mechanisms, one that is mediated by SREBF-1 and the other involving the direct binding of LXR/retinoid x receptor (RXR) heterodimer to the *Fasn* promoter [57]. It is, therefore, conceivable that the direct activation of *Fasn* expression by the LXR signaling pathway is behind our finding. In this regard, it is also important to point out that we observed a significant decrease in hepatic *Sdc1* expression in obese female *Sdc4^-/-^* mice. The expression of *Sdc1* is, in part, regulated by nuclear hormone receptors, such as the bile acid nuclear receptor farnesoid-X-receptor/RXR heterodimer [58]. Furthermore, it has been reported that *SDC4* SNP rs1981429 might function as a trans-eQTL SNP associated with the expression of the *RXRG* gene expression in the liver of Caucasians [59]. Taken together, these observations suggest that *Sdc4* deficiency could cause alterations in RXR subtype levels, which may subsequently affect the activation of the partners of RXRs involved in the regulation of lipid metabolism. Additional work using pharmacological tools and liver-specific conditional knockout mice is, however, needed to address this idea.

In conclusion, this study provides evidence that *Sdc4* plays a role in HFD-induced adiposity and that it may be involved in the sex differences in adiposity and metabolic outcomes resulting from high-fat feeding in mice. Considering that the relationship between body fat mass and various cardiometabolic risk factors also vary by sex in humans [60], our results may have a high relevance to human health. This is further supported by observations from a recent study, showing that serum levels of SDC4 were significantly associated with myocardial infarction in women [61]. In addition, high levels of *SDC4* gene expression are significantly associated with estrogen receptor-positive breast carcinomas [62], corroborating the notion of a relationship between SDC4 and estrogen/estrogen receptor signaling.

## Figures and Tables

**Table 1:** Repeated measures Analysis of Variance for percentage body weight changes in HFD-fed female and male *Sdc4^-/-^* and WT mice.

Source ^a^	*Df* *^b^*	*MS* *^c^*	*F* *^d^*	*P-Value*
Time	3.758	7,6926.32	157.99	<0.0001
Time x Genotype	3.758	279.68	0.57	0.6714
Time x Sex	3.758	2172.03	4.46	0.0031
Time x Genotype x Sex	3.758	419.45	0.86	0.4851
Error(Time)	82.672	486.92		

^a^ Source of variation. ^b^ Degrees of freedom. ^c^ Mean Squares computed from Type III Sums of Squares. ^d^ F-statistics based on the ratio of Mean Squares.

**Table 2:** Analysis of Variance for body composition and metabolic data in HFD-fed female and male *Sdc4^-/-^* and WT mice.

Phenotype	Source ^a^	*Df ^b^*	*MS ^c^*	*F ^d^*	*P-Value*	*Means ± SE*
%BFM	Sex	1	28.27	2.37	0.1377	F: 44.91 ± 2.04; M: 45.98 ± 0.41
	Genotype	1	290.98	24.43	<0.0001	WT: 42.20 ± 1.48; KO: 48.31 ± 0.60
	Genotype x Sex	1	210.86	17.70	0.0004	WTF: 37.62 ± 2.22; KOF: 50.13 ± 1.75 WTM: 45.48 ± 0.54; KOM: 46.48 ± 0.74
	Error	22	4.05			
%LBM	Sex	1	8.06	0.84	0.3691	F: 51.57 ± 2.04; M: 51.37 ± 0.41
	Genotype	1	246.52	25.72	<0.0001	WT: 54.55 ± 1.19; KO: 48.82 ± 0.57
	Genotype x Sex	1	152.68	15.93	0.0006	WTF: 58.06 ± 1.91; KOF: 46.94 ± 1.64 WTM: 52.03 ± 0.44; KOM: 50.71 ± 0.64
	Error	22	9.59			
TC (mg/dL)	Sex	1	37,771.05	100.23	<0.0001	F: 74.54 ± 5.91; M: 155.35 ± 7.21
	Genotype	1	497.05	1.32	0.2650	WT: 114.80 ± 15.85; KO: 119.36 ± 10.97
	Genotype x Sex	1	3165.53	8.40	0.0092	WTF: 57.61 ± 1.69; KOF: 90.48 ± 5.29 WTM: 162.45 ± 6.33; KOM: 148.24 ± 12.99
	Error	19	376.86			
TG (mg/dL)	Sex	1	1374.67	4.89	0.0401	F: 61.98 ± 5.78; M: 74.60 ± 5.90
	Genotype	1	1534.92	5.46	0.0312	WT: 61.44. ± 5.85; KO:75.05 ± 5.75
	Genotype x Sex	1	1627.66	5.80	0.0270	WTF: 41.32 ± 2.09; KOF: 75.76 ± 3.62 WTM: 74.85 ± 4.56; KOM: 74.34 ± 11.51
	Error	18	280.87			
Insulin (ng/dL)	Sex	1	15.21	188.34	<0.0001	F: 1.39 ± 0.07; M: 7.39 ± 0.87
	Genotype	1	0.01	0.09	0.7648	WT: 4.94 ± 1.33; KO:4.13 ± 0.91
	Genotype x Sex	1	0.23	2.81	0.1101	WTF: 1.22 ± 0.09; KOF: 1.53 ± 0.07 WTM: 8.05 ± 1.49; KOM: 6.73 ± 0.97
	Error	19	0.08			
Glucose(mg/dL)	Sex	1	0.20	7.61	0.0125	F:185.82 ± 12.24; M: 217.75 ± 11.43
	Genotype	1	0.02	0.87	0.3636	WT: 202.18 ± 18.00; KO: 202.75 ± 5.46
	Genotype x Sex	1	0.43	16.61	0.0006	WTF: 155.2 ± 19.23; KOF: 211.33 ± 4.07 WTM: 241.33 ± 16.36; KOM: 194.34 ± 9.23
	Error	19	0.026			
ISI	Sex	1	20.60	294.96	<0.0001	F: 32.40 ± 4.49; M: 4.86 ± 0.55
	Genotype	1	0.24	3.40	0.0810	WT: 22.90 ± 6.95; KO: 13.57 ± 2.45
	Genotype x Sex	1	1.57	22.41	0.0001	WTF: 45.57 ± 5.47; KOF: 21.43 ± 0.91 WTM: 4.01 ± 0.36; KOM: 5.71 ± 0.97
	Error	19	0.07			

^a^ Source of variation. ^b^ Degrees of freedom. ^c^ Mean Squares computed from Type III Sums of Squares. ^d^ F-statistics based on the ratio of Mean Squares. SE: Standard Error. %BFM: Percent body fat mass. %LBM; Percent lean body mass. TC: Total cholesterol. TG: Triglycerides. ISI: Insulin sensitivity index. F: Female. M: Male. KO: *Sdc4^-/-^* . WTF: Wild-type female; KOF: *Sdc4^-/-^* female. WTM: Wild-type male. KOM: *Sdc4^-/-^* male. Insulin, glucose, and ISI data were log_10_ transformed to fulfill the assumption of normality.

**Table 3:** Analysis of Variance for food intake, REE, and locomotor activity in HFD-fed female and male *Sdc4^-/-^* and WT mice.

Phenotype	Source *^a^*	*Df* *^b^*	*MS* *^c^*	*F* *^d^*	*P-Value*	*Means ± SE*
Food intake (g/day)	Sex	1	1.094	4.60	0.0438	F: 2.43 ± 0.15; M: 2.86 ± 0.10
	Genotype	1	1.615	6.79	0.0165	WT: 2.91 ± 0.14; KO: 2.39 ± 0.14
	Genotype x Sex	1	0.646	2.72	0.1141	WTF: 2.86 ± 0.27; KOF: 2.02 ± 0.20 WTM: 2.96 ± 0.08; KOM: 2.77 ± 0.19
	Error	21	0.238			
REE (kcal/24 h)	Sex	1	3.763	5.97	0.0230	F: 10.48 ± 0.25; M: 11.31 ± 0.20
	Genotype	1	0.518	0.82	0.3745	WT: 11.10 ± 0.24; KO: 10.78 ± 0.28
	Genotype x Sex	1	1.327	2.11	0.1608	WTF: 10.91 ± 0.39; KOF: 10.17 ± 0.29 WTM: 11.23 ± 0.31; KOM: 11.40 ± 0.27
	Error	22	0.630			
Locomotor activity (counts/24 h)	Sex	1	1.592	11.40	0.0027	F: 39419.64 ± 5330.50; M: 23350.49 ± 1654.00
	Genotype	1	0.329	2.36	0.1391	WT: 28073.67 ± 3668.21; KO: 33909.27 ± 5220.80
	Genotype x Sex	1	0.493	3.53	0.0736	WTF: 49817.96 ± 7849.61; KOF: 31992.27 ± 6155.85 WTM: 22545.92 ± 2161.63; KOM: 24155.06 ± 2639.25
	Error	22	0.140			

^a^ Source of variation. ^b^ Degrees of freedom. ^c^ Mean Squares computed from Type III Sums of Squares. ^d^ F-statistics based on the ratio of Mean Squares. SE: Standard Error. REE: Resting energy expenditure. F: Female. M: Male. KO: *Sdc4^-/-^*. WTF: Wild-type female; KOF: *Sdc4^-/-^* female. WTM: Wild-type male. KOM: *Sdc4^-/-^* male. Locomotor activity data were log_10_ transformed to fulfill the assumption of normality.

## Supplementary Materials

The following are available online at https://www.mdpi.com/2072-6643/11/11/2810/s1, Figure S1: Sdc4 deficiency does not alter transcript levels of other Sdc genes and Fasn gene in gonadal WAT from female mice fed a HFD for 14 weeks. Table S1: List of primers used in this study.
