# Supplementary material for: Genetic Deletion of Syndecan-4 Alters Body Composition, Metabolic Phenotypes, and the Function of Metabolic Tissues in Female Mice Fed A High-Fat Diet
Source: Nutrients. 2019 Nov 18;11(11):2810. doi: 10.3390/nu11112810 (PMC6893658; doi:10.3390/nu11112810)
Supplement: Supplementary file 1 [file nutrients-11-02810-s001.zip › nutrients-639082-supplementary.docx]

**Supplementary Material**

**Table S1. List of primers used in this study.**

| **Gene** | **Primer sequence** |
| --- | --- |
| *Adgre* | 5’-CTTTGGCTATGGGCTTCCAGTC-3’  3’-GCAAGGAGGACAGAGTTTATCGTC-5’ |
| *Cd11c* | 5'-CAAAATCTCCAACCCATGCT-3'  3’-TCTGGGAAGCCAAATACGAC-5’ |
| *Col1a1* | 5’-TGGCAACAAAGGAGACACTG-3’  3’-GGCTCCTCGTTTTCCTTCTT-5’ |
| *Col3a1* | 5’-ACCAAAAGGTGATGCTGGAC-3’  3’-GACCTCGTGCTCCAGTTAGC-5’ |
| *Col4a1* | 5’-CCAAAGGATCAGTTGGAGGA-3’  3’-CTCTCCTTTGGCTCCCTTCT-5’ |
| *Col5a1* | 5’-GGTCCCTGACACACCTCAGT-3’  3’-TGCTCCTCAGGAACCTCTGT-5’ |
| *Col6a1* | 5’-GGGACACGACACCTCTCAGT-3’  3’-TTGGCAGGAAATGACATTGA-5’ |
| *Mttp* | 5’-TCCAGGGTGGTCTAGCTAT-3’  3’-CCTTGTCCATCTGCATGCA-5’ |
| *ApoB* | 5’-TGCGGACGCCGTTACTGCTG-3’  3’-CAGATTTGGGGGACCTCCAG-5’ |
| *Srebf1* | 5’-GGAGCCATGGATTGCACATT-3’  3’-GCTTCCAGAGAGGAGGCCAG-5’ |
| *Fasn* | 5’-GCTGCGGAAACTTCAGGAAAT-3’  3’-AGAGACGTGTCACTCCTGGACTT-5’ |
| *Acaca* | 5’-TGACAGACTGATCGCAGAGAAAG-3’  3’-TGGAGAGCCCCACACACA-5’ |
| *Pparg* | 5’-GGAATCAGCTCTGTGGACCT-3’  3’-TGAGGCCTGTTGTAGAGCTG-5’ |
| *Dgat1* | 5’-TCCGTCCAGGGTGGTAGTG-3’  3’-TGAACAAAGAATCTTGCAGACGA-5’ |
| *Dgat2* | 5’-TTCCTGGCATAAGGCCCTATT-3’  3’-AGTCTATGGTGTCTCGGTTGAC-5’ |
| *G6pd* | 5’-TCGCGCTTGGATTCTACCTGCTA-3’  3’-TTGAAGAGGCTGGCAAAGGGTGTA-5’ |
| *Pck1* | 5’-TAGTGCCTGTGGGAAGACCAACTT-3’  3’-TTTGTCTTCACTGAGGTGCCAGGA-5’ |
| *Sdc1* | 5’- TTGGACCTAGATGGCCTATT-3’  3’- TAGCGGTCTCTATGGGTAAG-5’ |
| *Sdc2* | 5’- CAGAGCTGACATCCGATAAG-3’  5’- GCCTGAGGCAGAAGAATAG-3’ |
| *Sdc3* | 5’- ACCCGTTCTCTCCCTATAAC-3’  3’- CCCTCTCCTCCTTCTCTTATT-5’ |
| *Hprf* | 5’- GTTAAGCAGTACAGCCCCAAA-3’  3’- AGGGCATATCCAACAACAAACTT-5’ |
| *Tbp* | 5’- ACCCTTCACCAATGACTCCTATG-3’  3’- TGACTGCAGCAAATCGCTTGG-5’ |
| *Actb* | 5’- ccctgtatgcctctggtcgtaccac-3’  3’- gccagccaggtccagacgcaggatg-3’ |


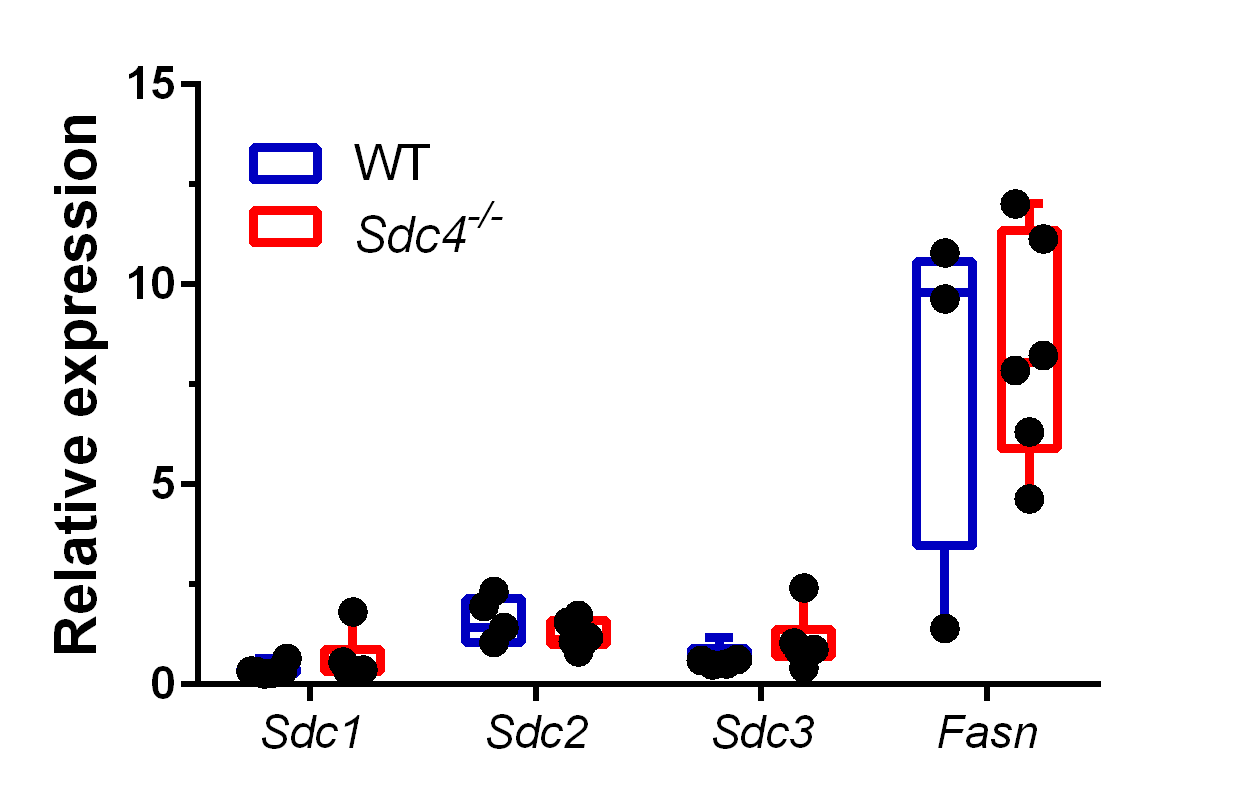


**Figure S1. *Sdc4* deficiency does not alter transcript levels of other *Sdc* genes and *Fasn* gene in gonadal WAT from female mice fed a HFD for 14 weeks.**  (A-D) Box and whiskers plots denote individual data points separated by a line representing the group median. Each individual value is plotted as a dot superimposed on the boxplots. Transcript levels of each target gene were normalized to *Hprt*, *Actb*, and *Tbp* genes.
